# Precision medicine: a few thoughts from 2022

**DOI:** 10.1093/pcmedi/pbac003

**Published:** 2022-02-12

**Authors:** Sherman Morton Weissman

**Affiliations:** Department of Genetics, Yale School of Medicine, New Haven, CT 06520-8005, USA

Happy holiday season and a good new year for all the readers! It's been a year for substantial progress in some aspects of immune therapy. In general, precision medicine-based therapies have focused more on cancer, but at the same time, on other diseases like inflammatory bowel disease. We're learning to divide diseases into those that might be susceptible in individuals to cytokine or antibody therapy and those that are resistant and for which new therapies have to be developed. One of the more dramatic developments is the discovery of ways to target at least some specific mutations of the current substantial portion of cancers, using small molecule drugs as inhibitors. I'm specifically thinking of building drugs to inhibit certain RAS mutations that are recurrent drivers of common types of cancer. These drugs should be powerful, and offer a leading example of targeting “untargetable” proteins.

Now there is the development of methods for predicting protein structure, even for proteins that have not been investigated experimentally. This methodology expands the range of targets for which small molecule treatments can be designed. This is one very exciting area that has emerged within the last few years.

At the same time, there's continuing success in classifying cancers, not just on the basis of cell type, but on the basis of the specific types of molecular pathology of individual tumors, such as some types of colon tumors, for which we have to develop new therapies, as opposed to those in which enabling the natural immune response is showing great usefulness. In general, a pressing challenge is to develop approaches for tumors that have developed other protective mechanisms that excluded T cells from tumors. The ultimate goal, of course, is to develop therapies that target any mutations of each individual tumor. Immune therapy works so well against a portion of tumors for which there are good antigens at the cell surface. This encourages one to try to develop more ways of getting cells to express more of their mutations as antigens that are available on the cell surface. So manipulating the immune system in various ways continues to be promising for full gene therapy. This is one approach, the approach of taking advantage of those tumors that infiltrate lymphocytes where the body has already found targets that are recognized by tumor infiltrating T cells. We are on the way to producing tumor infiltrating lymphocytes for personalized cancer treatment.

Cutting-edge technology such as tumor cell sequencing at the single-cell level is providing new knowledge about tumor biology and susceptibility. These methodologies are developing very rapidly with increasing integration of single-cell genomics, the special genomics of individual tumor cells, with imaging the genome using 3D methods. There are an increasing number of approaches we cannot begin to touch on in this brief talk, and increasing hope for future therapy for a larger number of tumors, but there is still a requirement for new approaches for the most resistant tumors and most advanced tumor types.

In addition to tumors, autoimmune diseases would seem to be an area one should be able to build therapies for based on the particular immune response. For instance, in some cases, if it's a monoclonal antibody that the body is producing, then developing mechanisms to specifically attack cells producing an antibody should offer more hope for the future. There are many ways to approach immune therapy, so good luck to investigators. And let's hope that governments recognize the value of supporting this kind of work and the value of encouraging international collaborations to cope with international problems, because diseases don't recognize country boundaries or protocol systems. They recognize people.

Hope *Precision Clinical Medicine* serves as a broader platform for all investigators to tackle the medical challenges and share the most updated knowledge for diseases and treatments.

Happy New Year!

Sherman Weissman

Yale School of Medicine

